# Retinal vascular impairment in Wolfram syndrome: an optical coherence tomography angiography study

**DOI:** 10.1038/s41598-022-06150-6

**Published:** 2022-02-08

**Authors:** Marco Battista, Maria Lucia Cascavilla, Domenico Grosso, Enrico Borrelli, Giulio Frontino, Giulia Amore, Michele Carbonelli, Riccardo Bonfanti, Andrea Rigamonti, Costanza Barresi, Chiara Viganò, Beatrice Tombolini, Anna Crepaldi, Marina Montemagni, Chiara La Morgia, Francesco Bandello, Piero Barboni

**Affiliations:** 1 Department of Ophthalmology, IRCCS Ospedale San Raffaele, University Vita-Salute, Via Olgettina 60, Milan, Italy; 2 Diabetes Research Institute, IRCCS Ospedale San Raffaele, University Vita-Salute, Milan, Italy; 3 Pediatric Department, IRCCS Ospedale San Raffaele, Milan, Italy; 4 Unit of Neurology, Department of Biomedical and NeuroMotor Sciences (DIBINEM), University of Bologna, Bologna, Italy; 5 Department of Ophthalmology, Policlinico Federico II, Napoli, Italy; 6 UOC Clinica Neurologica, IRCCS Istituto Delle Scienze Neurologiche Di Bologna, Bologna, Italy

**Keywords:** Genetics, Diseases, Medical research

## Abstract

To evaluate differences in macular and optic disc circulation in patients affected by Wolfram Syndrome (WS) employing optical coherence tomography-angiography (OCTA) imaging. In this retrospective study, 18 eyes from 10 WS patients, 16 eyes of 8 patients affected by type I diabetes and 17 eyes from 17 healthy controls were enrolled. All patients were imaged through OCT and OCTA and vascular parameters, as perfusion density (PD) and vessel length density (VLD) were measured. OCTA showed reduced PD in WS patients at the macular superficial capillary plexus (SCP, 27.8 ± 5.3%), deep vascular complex (DVC, 33.2 ± 1.9%) and optic nerve head (ONH, 21.2 ± 9.1%) compared to both diabetic patients (SCP 33.9 ± 1.9%, *P* < 0.0001; DVC 33.2 ± 0.7%, *P* = 1.0; ONH 33.9 ± 1.3, *P* < 0.0001) and healthy controls (SCP 31.6 ± 2.5, *P* = 0.002; DVC 34.0 ± 0.7%, *P* = 0.089; ONH 34.6 ± 0.8%, *P* < 0.0001). Similarly, VLD was lower in WS patients at the SCP (10.9 ± 2.7%) and ONH levels (7.5 ± 4.1%) compared to diabetic patients (SCP 13.8 ± 1.2%, *P* = 0.001; DVC 13.8 ± 0.2%, *P* < 0.0001; ONH 13.0 ± 0.7%, *P* =  < 0.0001), but higher in DVC (15.7 ± 1.2%, *P* < 0.0001). Furthermore, VLD was lower in WS patients in all the vascular parameters compared to controls (SCP 13.8 ± 1.5%, *P* < 0.0001; DVC 17.3 ± 0.6%, *P* < 0.0001; ONH 15.7 ± 0.5%, *P* < 0.0001). A significant microvasculature impairment in the macular SCP and ONH microvasculature was demonstrated in eyes affected by WS. Microvascular impairment may be considered a fundamental component of the neurodegenerative changes in WS.

## Introduction

Wolfram syndrome (WS) is a rare autosomal recessive progressive disease^1,2^ caused largely by mutations in the WFS1 gene^3,4^ and, less commonly, in the CISD2 gene (WS type 2)^5^. WS is characterized by childhood onset insulin-dependent diabetes mellitus and optic atrophy associated in a variable number of cases to diabetes insipidus, sensorineural hearing loss and neurodegeneration^6^. Prognosis of WS is currently poor as most of patients die prematurely with severe neurological disabilities^7,8^.

WFS1 gene encodes for wolframine, a transmembraneous protein in the endoplasmic reticulum (ER)^3^ and is highly expressed in brain tissue^9^, pancreatic β-cells, and in the heart^10^. Of note, loss of function mutations of WFS1 gene triggers a cascade of ER and mitochondrial dysfunction that ultimately leads to apoptosis and cellular death^11,12^.

Wolframin is also expressed in retina, specifically in retinal ganglion cells (RGCs) and in non-myelinated portion of the optic nerve^13,14^. In fact, optic atrophy is a constant feature in WS, which commonly presents in patient as a gradual visual acuity loss, color vision deficiency and central scotomas on visual field examination^15^.

Optical coherence tomography angiography (OCTA) is a recently introduced technique which allows the non-invasive evaluation of the retinal, choroidal and ONH vascular circulations avoiding the dye injection-related adverse events^16^. Based on recent OCTA studies in literature, the superficial retinal capillary plexus (SCP) is made up of macular vessels interposed between the retinal nerve fiber layer and ganglion cell layers, as the deep retinal capillary plexus (DCP) is detected at the outer border of the inner nuclear layer. SCP and DCP act as the main retinal capillary networks in the sustenance of the inner and middle layers of the retina^17,18^. For the optic nerve head analysis on OCTA, the disc-area is conventionally divided into 3 layers, namely the optic nerve head (ONH), radial peripapillary capillary (RPC) and choroid layers^19^^,^^20^ Recent studies have reported the density of the retinal microvascular networks in normal and pathologic states using OCTA in adults and children^21–26^.

Although the prominent feature of WS is optic atrophy, vascular impairment of the retina and ONH has been previously described and analyzed by fluorescein angiography (FA)^27^. The latter study did not differentiate vascular lesion due to diabetic retinopathy from vascular changes intrinsically related to WS. Recently, Asanad and coauthors^28^ found a decline in the vascular network of the ONH and RPC layers, most pronounced in the temporal region. These findings are comparable with vascular alteration in other optic neuropathy, such as Leber’s hereditary optic neuropathy (LHON)^29^ and dominant optic atrophy (DOA)^30^.

However, to the best of our knowledge, description of morphological feature of macular microvasculature using OCTA has not been performed in WS. A precise vascular assessment of this territory could give new insights on the capillary remodeling which occurs along with the structural impairment.

The aim of this study was to investigate quantitative differences in macular retinal and optic disc circulation of patients affected by WS, using OCTA analysis.

## Methods

In this retrospective study, patients with a clinical and molecularly confirmed diagnosis of WS were enrolled from the neuro-ophthalmology clinic at the San Raffaele Hospital. The study adhered to the tenets of the Declaration of Helsinki and the Health Insurance Portability and Accountability Act. Written informed consent was obtained from all subjects or their legal tutor prior to enrollment in the study and it was authorized by the San Raffaele Ethics Committee.

In order to perform comparisons, two additional groups were also enrolled: (i) a second group with patients affected by type I diabetes mellitus who were enrolled at the pediatric department at the San Raffaele Hospital, and (ii) a control group of healthy subjects.

All patients and controls were enrolled between March 2020 and February 2021 and received a complete ophthalmologic examination, including the measurement of best-corrected visual acuity (BCVA), intraocular pressure (IOP), and dilated ophthalmoscopy. Best corrected visual acuity (BCVA) was assessed using Snellen charts and listed as logMAR equivalents.

For the first two groups, exclusion criteria were the presence of any retinal pathology and/or optic nerve disease other than, respectively, WS and diabetic retinopathy.

All control subjects were needed to have a refractive error of less than 3 diopters and no evidence of retinal disease or ocular media opacity after evaluation by dilated fundus examination and OCTA.

All patients and controls underwent swept source (SS) OCT and OCTA imaging using the DRI Triton device (Topcon; Tokyo, Japan). Volumetric scans of the RNFL and GC-IPL were obtained through a three-dimensional scanning procedure performed with a 6 × 6 mm raster scan centered on the fovea and optic disc composed of 256 B-scans, each consisting of 256 A-scans. Poor-quality images with artifacts, such as motion artifacts, blinking or segmentation failure, were not included in the analysis. The report generated by the machine gives the color image of central macular with image centered at the fovea. The central macular thickness (CMT) was considered as the 1 mm concentric circle from the center of fovea. Average RNFL thickness was measured in a 3.4 mm circle centered around the optic disc, whereas average GC-IPL thickness was measured in a 6mmx6mm macular region. The software automatically calculated the average and sectorial RNFL (S, N, I, and T) thicknesses and GCC mean. The segmentation was checked and manually adjusted if the fully automated algorithm failed to select the correct boundaries.

**Figure 1:**
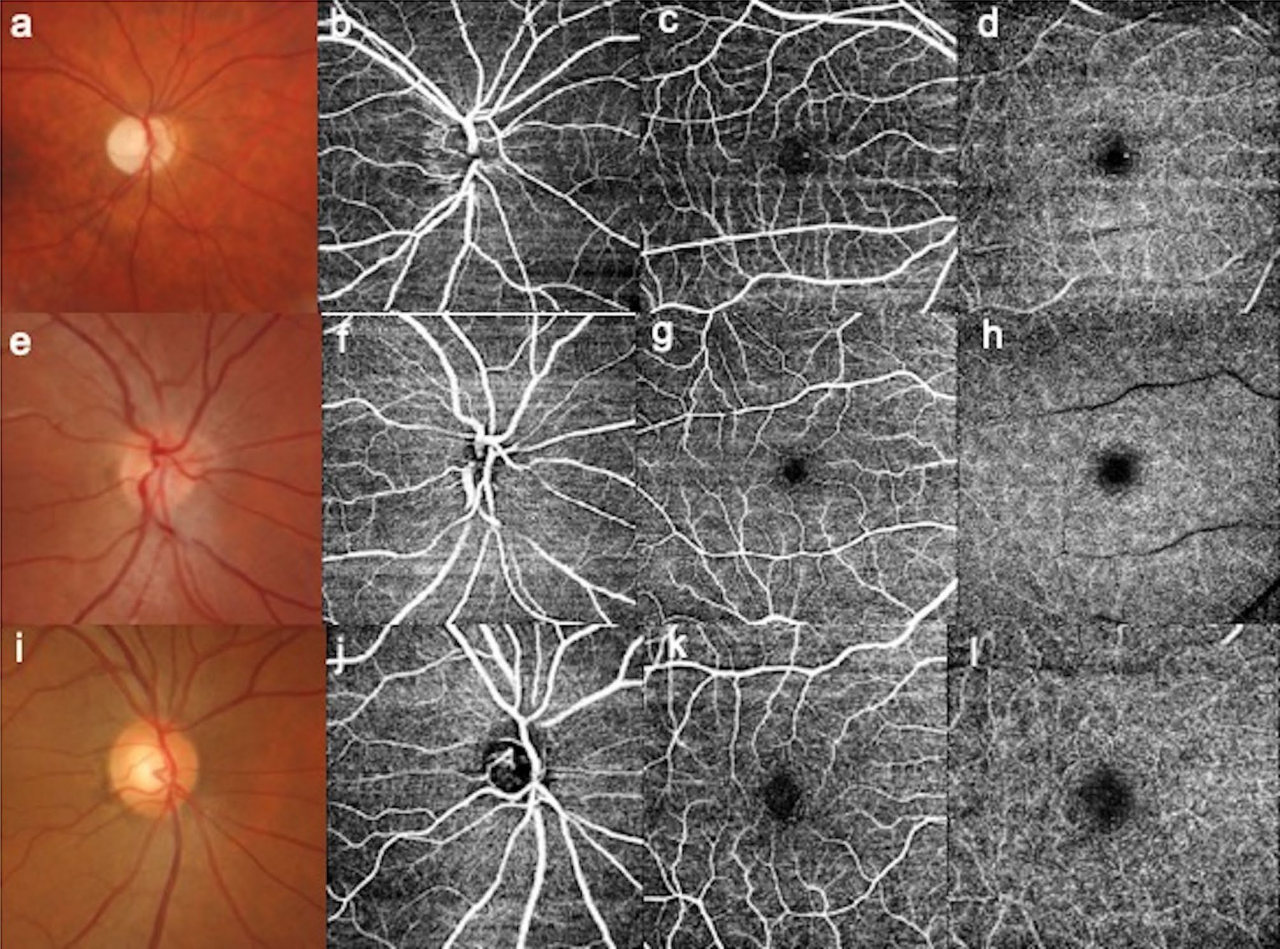
Optic nerve head photography and OCTA images of the optic disc area, macular superficial and deep capillary plexus. Color fundus photography of the optic disc (**a**), optic nerve head (ONH; **b**), macular superficial capillary plexus (SCP; **c**) and deep vascular complex (DVC; **d**) imaged by optical coherence tomography-angiography (OCTA) in a patient affected by Wolfram Syndrome; color fundus photography of the optic disc (**e**), ONH (**f**) and macular SCP (**g**), DVC (**h**) in a young patient affected by type I diabetes; ONH color photography (**i**) and OCT-A acquisitions centered on the optic disc (**j**) and macula (**k**, **l**) in a healthy patient.

OCTA images were obtained with a scan area of 6mmx6mm centered at the fovea and optic disc. En-face images of the vascular structure in the superficial macular capillary plexus (SCP), deep vascular complex (DVC, grouping middle capillary plexus and deep capillary plexus) and superficial vascular layer of the optic disc region (ONH) were automatically generated (Fig. 1).

The main outcome measures for this study were SCP perfusion density (PD) and vessel length density (VLD); PD and VLD in the DVC; PD and VDL in the peripapillary area.

**Figure 2:**
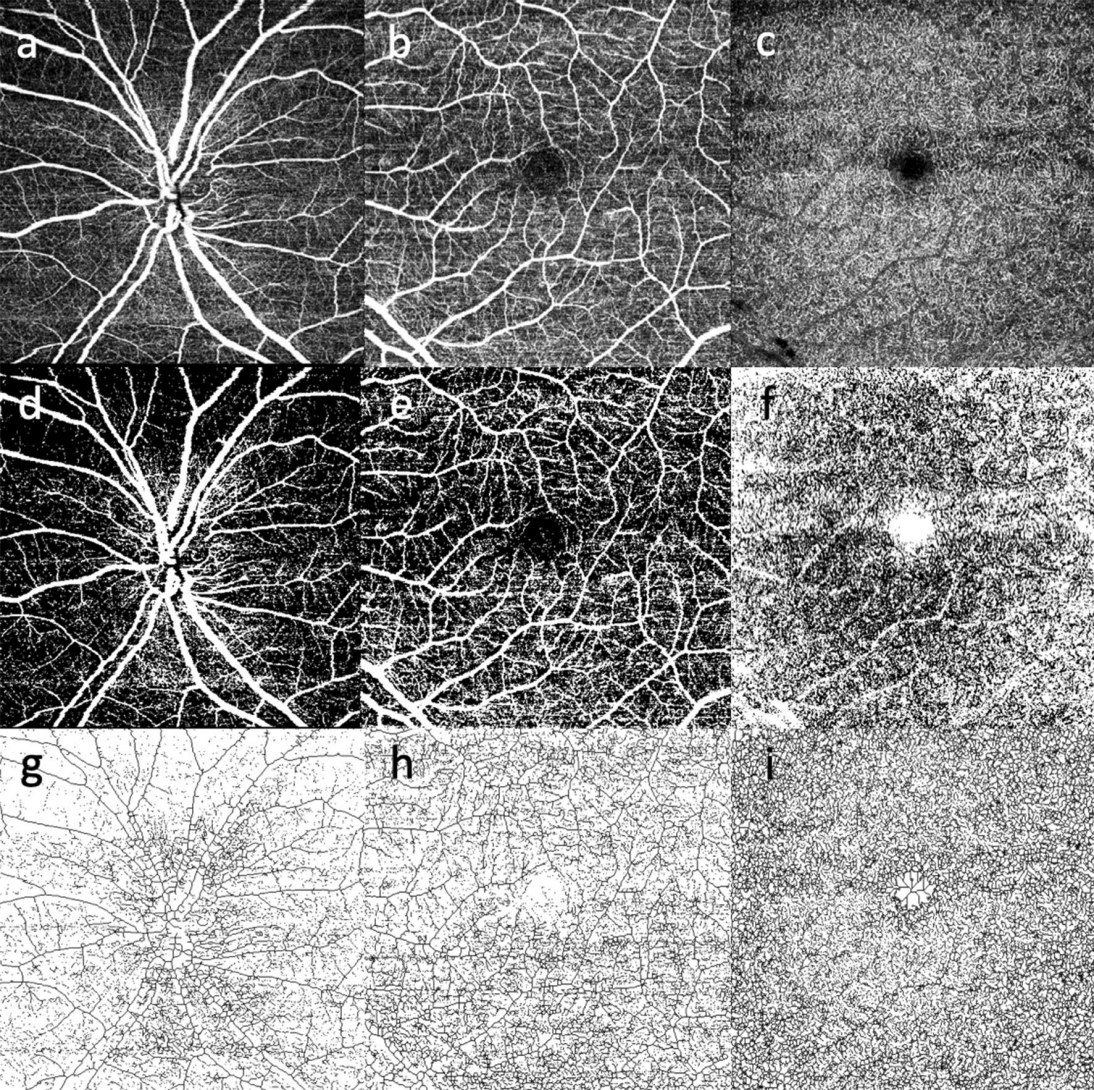
Explanatory case processed to obtain binarized and skeletonized images for perfusion density and vessel length density calculation. Optic nerve head (ONH; **a**), superficial capillary plexus (SCP; **b**) and deep vascular complex (DVC; **c**) imaged through optical coherence tomography angiography (OCTA) in a patient affected by optic atrophy in Wolfram Syndrome. The same panel after post-acquisition binarization (**d**; **e**; **f**) and skeletonization process (**g**; **h**; **i**).

Two graders (E.B. and D.G.), masked to the patients’ information, checked the quality of all images and only good quality images were considered for the analysis. SCP, DVC and ONH scans were exported and processed through Fiji-ImageJ software (National Institutes of Health, Bethesda, Maryland, USA; available at http://rsb.info.nih.gov/ij/index.html). To calculate the PD, a previously described thresholding algorithm was applied to the SCP, DVC and ONH en face images to create binary images (Fig. 2)^31^. The PD was calculated as the proportion of the number of pixels over the threshold divided by the total pixels number in the analyzed area. The VLD, instead, was defined as the total length of the perfused vasculature divided by the total pixel number in the reference area.

### Statistical analysis

Statistical calculations were performed using Statistical Package for Social Sciences (version 20.0, SPSS Inc., Chicago, IL, USA).

To detect departures from normal distribution, a Shapiro–Wilk’s test was performed for all variables. Means and standard deviation (SD) were computed for all quantitative variables. Continuous variables were compared by conducting a one-way analysis of covariance (ANCOVA) with Bonferroni post-hoc test, by introducing age as covariate. The false discovery rate correction (FDR) was used to control the family-wise type I error rate, and an FDR adjusted *P* value < 0.05 was determined to be statistically significant.

Pearson’s chi-squared correlation was performed to evaluate the linear correlations.

## Results

In the first group of WS patients (n = 10), 18 eyes were considered for the analysis (two eyes were excluded for poor quality images). Sixteen eyes from 8 patients affected by type I diabetes were enrolled in the second group. Seventeen eyes from 17 healthy subjects were included in the control group and only one eye per every healthy control was randomly selected. Demographic and clinical characteristics of the three groups were summarized in Table 1.

**Table 1:** Main demographic and clinical features of the three groups analyzed: patients affected by Wolfram Syndrome (WS), patients affected by type I diabetes mellitus and healthy controls.

WS	DM	Controls
**Age, years (Mean ± SD)**		
24 ± 11	13.29 ± 2	40.8 ± 13
	< 0.0001^a^	< 0.0001^b^
		1.0^c^
**Sex**		
3/10 M	5/8 M	6/17 M
**BCVA, LogMAR (Mean ± SD)**		
0.44 ± 0.29	0.03 ± 0.05	0.03 ± 0.04
	< 0.0001^a^	< 0.0001^b^
		1.0^c^
**CMT, (Mean ± SD)**		
227.6 ± 6.1	235.8 ± 8.7	230.8 ± 19.3
	0.041^a^	0.127^b^
		0.495^c^
**RNFL thickness, µm (Mean ± SD)**		
47.1 ± 11.9	102.8 ± 15.7	107.9 ± 9.7
	< 0.0001^a^	< 0.0001^b^
		0.695^c^
**GCC thickness, µm (Mean ± SD)**		
44.9 ± 5.1	72.6 ± 5.2	70.2 ± 4.6
	< 0.0001^a^	< 0.0001^b^
		0.551^c^

Values were compared with one-way analysis of covariance (ANCOVA) with age as covariate.

^a^DM versus WS.

^b^Controls versus WS.

^c^Controls versus DM.

*WS* Wolfram Syndrome, *DM* diabetes mellitus, *SD* standard deviation, *BCVA* best-corrected visual acuity, *CMT* central macular thickness, *RNFL* retinal nerve fiber layer, *GCC* ganglion cell complex.

Disease duration was 14.1 years (range 4–29 years) for WS patients and type I diabetes was the onset manifestation of the syndrome for 8/10 patients. Optic atrophy was the first manifestation of WS in 2 patients without diabetes. Optic atrophy was observed in all the enrolled patients and the mean onset age was 10.8 ± 4.1 years. Urological dysfunction was detected in 2 patients (atonic bladder), diabetes insipidus in one patient. Diabetes duration was 3.7 ± 1.2 years in the second study group; no signs of diabetic retinopathy were detected during the ophthalmic evaluation. From OCT analysis, the average CMT was 227.6 ± 6.1 µm in WS eyes, 235.8 ± 8.7 µm in diabetic patients and 230.8 ± 19.3 µm in the control group.

The average RNFL and GCC thickness was significantly reduced in WS eyes compared to diabetic patients and healthy subjects. (*P* < 0.0001) (Table 1). The PD was lower in WS patients at the macular SCP (27.8 ± 5.3%), and ONH (21.2 ± 9.1%) levels, if compared to both diabetic patients (SCP 33.9 ± 1.9%, *P* < 0.0001; ONH 33.9 ± 1.3, *P* < 0.0001) and controls (SCP 31.6 ± 2.5, *P* = 0.002; ONH 34.6 ± 0.8%, *P* < 0.0001). Similarly, VLD was lower in WS patients at the SCP (10.9 ± 2.7%) and ONH levels (7.5 ± 4.1%), compared to diabetic patients (SCP 13.8 ± 1.2%, *P* = 0.001; DVC 13.8 ± 0.2%, *P* < 0.0001; ONH 13.0 ± 0.7%, *P* =  < 0.0001), but higher in DVC (15.7 ± 1.2%,  *P * < 0.0001). Furthermore, VLD was lower in WS patients in all the considered parameters compared to controls (SCP 13.8 ± 1.5%, *P* < 0.0001; DVC 17.3 ± 0.6%, *P *< 0.0001; ONH 15.7 ± 0.5%, *P* < 0.0001). The OCTA parameters are summarized in Table 2.

**Table 2:** Optical coherence tomography-angiography vascular features of the three cohorts, expressed as perfusion density and vessel length density.

WS	DM	Controls
**Macular SCP PD % (Mean ± SD)**		
27.8 ± 5.3	33.9 ± 1.9	31.6 ± 2.5
	< 0.0001^a^	0.002^b^
		0.279^c^
**Macular DVC PD % (Mean ± SD)**		
33.2 ± 1.9	33.2 ± 0.7	34.0 ± 0.7
	1.0^a^	0.089^b^
		0.312^c^
**ONH PD % (Mean ± SD)**		
21.2 ± 9.1	33.9 ± 1.3	34.6 ± 0.8
	< 0.0001^a^	< 0.0001^b^
		0.725^c^
**Macular SCP VLD % (Mean ± SD)**		
10.9 ± 2.7	13.8 ± 1.2	13.8 ± 1.5
	0.001^a^	< 0.0001^b^
		1.0^c^
**Macular DVC VLD % (Mean ± SD)**		
15.7 ± 1.2	13.8 ± 0.2	17.3 ± 0.6
	< 0.0001^a^	< 0.0001^b^
		< 0.0001^c^
**ONH VLD % (Mean ± SD)**		
7.5 ± 4.1	13.0 ± 0.7	15.7 ± 0.5
	< 0.0001^a^	< 0.0001^b^
		0.159^c^

Values were compared with one-way analysis of covariance (ANCOVA) with age as covariate.

^a^DM versus WS.

^b^Controls versus WS.

^c^Controls versus DM.

*SD* standard deviation, *SCP* superficial capillary plexus, *PD* perfusion density, *DVC* deep vascular complex, *ONH* optic nerve head, *VLD* vessel length density.

## Discussion

Our study revealed for the first time by OCTA a significant vascular impairment in the SCP in the macular and in the optic nerve area in a cohort of WS patients. Whereas, the DVC remained relatively spared in WS mainly in comparison with in diabetes group which was significantly reduced.

WS, originally described as a mitochondrial disorder^32^, is nowadays recognized to be caused by a primary impairment of the endoplasmic reticulum (ER)^9^. ER works closely with mitochondria and this interaction is mediated by mitochondria-associated membranes (MAMs)^33^. From studies on human cells, it appears that the dysregulation of Ca^2+^ homeostasis and the impaired MAMs formation lead to a reduced tethering between ER-mitochondria and ultimately impacting on the efficacy of cellular mitochondrial respiration in energy production^34,35^. The greater damage is observed on tissues with high energy demand as the optic nerve. The clinical finding of ONH pallor is the classical reflex of the primary optic atrophy and the grade of ONH involvement is quantified by OCT. The severe reduction of RNFL and GCC found in our WS cohort is in accordance with previous studies^36–38^. In particular, many studies highlighted that the inferior quadrants is usually the more involved in inherited optic neuropathies^39,40^.

DOA is the archetype of the mitochondrial optic neuropathies affecting the small axons of the papillo-macular bundle. Balducci and colleagues confirmed by OCT-A a significant microvascular impairment in the optic nerve area, in particular in the temporal sector^41^. However, the authors concluded that it is not possible to discern whether the vascular impairment is primary or secondary to the nerve fiber reduction observed in the progression of the disease^41^.

This is the first study employing OCTA in a cohort of WS patients. Moreover, the results of the WS cohort were compared to two control groups: a cohort of type I diabetic patients and a cohort of healthy controls. Furthermore, the absence of signs of diabetic retinopathy in both WS and diabetes group confirms that the microvascular changes are independent from diabetes-related vascular changes. In a case-report from Asanad, a peripapillary reduction in the superficial plexus of the peripapillary area was observed^28^.

In our study, the microvasculature of SCP and ONH was consistently impaired with a reduction of the vascular texture and a depletion of the vascular branching. Conversely, the DVC remained relatively spared with significant reduction of VLD and not of PD. DVC comprised the intermediate retinal capillary plexus and the deep capillary plexus and many studies highlighted its vulnerability in several vascular and non-vascular retinal diseases, ranging from early diabetic retinopathy to retinal dystrophies^42,43^. Conversely, this finding could confirm that in WS the vascular damage is secondary to the significant thickness reduction in the RNFL. However, we demonstrated that the microvascular damage is also located in the macular SCP and, for this aspect, the previous explanation is not sufficient. We speculated that the remarkable damage to the GCC observed, with the consequent cell death, may lead to a reduction in the energy demand and a secondary reduction, firstly functional than anatomical, in vascular flow. However, a structural support loss for vessels, due to ganglion cells degeneration, cannot be ruled out.

Type I diabetes mellitus is one of the earliest manifestations in WS^6^. Microvascular changes in retinal and ONH vascular plexus are a well-known feature in patients with and without diabetic retinopathy from long-term type I diabetes and several OCTA studies have confirmed this evidence^44,45^. From a recent metanalysis, macular PD of the whole-image, parafoveal, perifoveal region of both SCP and DCP resulted all decreased in eyes of diabetic patients without diabetic retinopathy^46^. Moreover, in type I patients capillary density in both the SCP and DCP seem to decrease with both age and increasing duration of disease^47^. VLD is a vascular parameter independent from the thickness of retinal vessels enabling a one-dimension analysis of retinal microvasculature^48^. In our series, VLD is similarly reduced both in SCP and DCP in eyes affected by diabetic retinopathy^49^. In our group of type I diabetic individuals, we found a significant VLD reduction in the DVC. For this reason, it appears very unlikely that the vascular impairment observed in WS eyes, mostly located in the macular SCP and ONH area, is due to diabetes, also because no signs of diabetic retinopathy were detected in WS patients; whereas we cannot exclude the role of diabetes in the macular DVC reduction in WS group.

This study has several limitations as the retrospective nature and the enrollment of both eyes for the majority of WS patients. However, it is important to highlight that WS is a very rare disease and patients have, unfortunately, a low life expectancy. A correlation analysis between BCVA and OCTA parameters was not made for the small patients’ number and the inconsistency of the results obtained. We underline that, in this setting, BCVA could be misinterpreted and visual reservoir could be better assessed through perimetric exams. The angiographic analysis was performed through a single OCTA device and we cannot exclude a different outcome with a diverse instrumental choice.

In conclusion, we demonstrated superficial microvasculature impairment both in the macular area and in the peripapillary area in eyes affected by WS. It is possible to hypothesize that the neurodegenerative damage of the optic nerve appears strictly connected with a vascular supply change, even though we cannot be sure of the direction of this interaction. These data include the vascular component in the pathophysiology adding a new feature to this complex disease.
